# Volume Expansion with Albumin Compared to Gelofusine in Children with Severe Malaria: Results of a Controlled Trial 

**DOI:** 10.1371/journal.pctr.0010021

**Published:** 2006-09-15

**Authors:** Samuel Akech, Samson Gwer, Richard Idro, Greg Fegan, Alice C Eziefula, Charles R. J. C Newton, Michael Levin, Kathryn Maitland

**Affiliations:** 1 Kenya Medical Research Institute/Wellcome Trust Research Programme, Centre for Geographic Medicine Research–Coast, Kilifi, Kenya; 2 Infectious Diseases Epidemiology Unit, London School of Hygiene and Tropical Medicine, London, United Kingdom; 3 Neurosciences Unit, Institute of Child Health, London, United Kingdom; 4 Department of Paediatrics and Wellcome Trust Centre for Clinical Tropical Medicine, Faculty of Medicine, Imperial College, London, United Kingdom

## Abstract

**Objectives:**

Previous studies have shown that in children with severe malaria, resuscitation with albumin infusion results in a lower mortality than resuscitation with saline infusion. Whether the apparent benefit of albumin is due solely to its colloidal properties, and thus might also be achieved with other synthetic colloids, or due to the many other unique physiological properties of albumin is unknown. As albumin is costly and not readily available in Africa, examination of more affordable colloids is warranted. In order to inform the design of definitive phase III trials we compared volume expansion with Gelofusine (succinylated modified fluid gelatin 4% intravenous infusion) with albumin.

**Design:**

This study was a phase II safety and efficacy study.

**Setting:**

The study was conducted at Kilifi District Hospital, Kenya.

**Participants:**

The participants were children admitted with severe falciparum malaria (impaired consciousness or deep breathing), metabolic acidosis (base deficit > 8 mmol/l), and clinical features of shock.

**Interventions:**

The interventions were volume resuscitation with either 4.5% human albumin solution or Gelofusine.

**Outcome Measures:**

Primary endpoints were the resolution of shock and acidosis; secondary endpoints were in-hospital mortality and adverse events including neurological sequelae.

**Results:**

A total of 88 children were enrolled: 44 received Gelofusine and 44 received albumin. There was no significant difference in the resolution of shock or acidosis between the groups. Whilst no participant developed pulmonary oedema or fluid overload, fatal neurological events were more common in the group receiving gelatin-based intervention fluids. Mortality was lower in patients receiving albumin (1/44; 2.3%) than in those treated with Gelofusine (7/44; 16%) by intention to treat (Fisher's exact test, *p =* 0.06), or 1/40 (2.5%) and 4/40 (10%), respectively, for those treated per protocol (*p =* 0.36). Meta-analysis of published trials to provide a summary estimate of the effect of albumin on mortality showed a pooled relative risk of death with albumin administration of 0.19 (95% confidence interval 0.06–0.59; *p =* 0.004 compared to other fluid boluses).

**Conclusions:**

In children with severe malaria, we have shown a consistent survival benefit of receiving albumin infusion compared to other resuscitation fluids, despite comparable effects on the resolution of acidosis and shock. The lack of similar mortality benefit from Gelofusine suggests that the mechanism may involve a specific neuroprotective effect of albumin, rather than solely the effect of the administered colloid. Further exploration of the benefits of albumin is warranted in larger clinical trials.

## Editorial Commentary

**Background:** In Africa, children admitted to hospital with severe malaria are at high risk of death even though effective malaria treatment is available. Death typically occurs during a narrow time window after admission and before antimalarial treatments can start working. Acidosis (excessive acidity of the blood) is thought to predict death, but it is not clear how acidosis arises. One possibility is that hypovolemia (lowered blood fluid volume) is important, which would normally require urgent resuscitation with fluids. However, there is little evidence on what type of fluid should be given. In the trial reported here, carried out in Kenya's Kilifi District Hospital between 2004 and 2006, 88 children admitted with severe malaria were assigned to receive either albumin solution (a colloid solution made from blood protein) or Gelofusine (a synthetic colloid). The primary outcomes that the researchers were interested in were correction of shock and acidosis in the blood after 8 h. However, the researchers also looked at death rate in hospital and adverse events after treatment.

**What this trial shows:** The investigators found no significant differences in the primary outcomes (correction of shock and acidosis in the blood 8 h after fluids were started) between children given Gelofusine and those given albumin. However, they did see a difference in death rates between children given Gelofusine and those given albumin. Death rates in hospital were lower in the group given albumin, and this was statistically significant. The researchers then combined the data on death rates from this trial with data from two other trials with an albumin arm. This combined analysis also supported the suggestion that death rates with albumin were lower than with other fluids, either Gelofusine or salt solution.

**Strengths and limitations:** There is currently very little evidence from trials to guide the initial management of fluids in children with severe malaria. The results from this trial indicate that further research is a priority. However, the actual findings from this trial must be tested in larger trials that recruit enough children to establish reliably whether there is a difference in death rate between albumin treatment and treatment with other fluids. This trial was not originally planned to find a clinically relevant difference in death rate, and therefore does not definitively answer that question. Further trials would also need to use a random method to assign participants to the different treatments, rather than alternate blocks (as in this trial). A random method ensures greater comparability of the two groups in the trial, and reduces the chance of selection bias (where assignment of patients to different treatments can be distorted during the enrollment process).

**Contribution to the evidence:** This study adds data suggesting that fluid resuscitation with albumin solution, as compared to Gelofusine, may reduce the chance of death in children with severe malaria. However, this finding is not definitive and would need to be examined in further carefully controlled trials. If the finding is supported by further research, then a solution to the problems of high cost and limited availability of albumin will need to be found.

## INTRODUCTION

Falciparum malaria remains a major cause of morbidity and mortality in African children, resulting in nearly 1 million deaths annually [1]. For children presenting to hospital with severe malaria, in-patient mortality remains high despite effective antimalarial treatment, especially among those presenting in coma, most deaths occurring within hours of admission. Metabolic acidosis, rather than impaired consciousness, has been shown to be the best independent predictor of death and thus represents an ideal target for a directed intervention aimed at improving early mortality [2]. In critically ill children, regardless of the underlying causative disease, the aetiology of metabolic acidosis is most frequently impaired perfusion [3]. Owing to a lower cardiovascular reserve relative to adults, shock states develop frequently in severe illness in children, and are generally treated by volume expansion, to optimise tissue and organ perfusion [4,5]. Volume resuscitation would therefore seem a logical intervention, in severe malaria, if hypovolaemia were aetiologically important. However, in most hospitals the use of volume expansion in children with severe malaria is uncommon, and may be actively discouraged because of concerns over its potential to cause pulmonary or cerebral oedema and because of the lack of definitive evidence to support its benefit.

Over the past few years we have provided new, clear evidence that hypovolaemia is common in children with severe malaria complicated by acidosis [6,7]. Volume expansion would generally be expected to be beneficial—if the acidosis is due to volume depletion. Nevertheless, in conditions where the integrity of the blood–brain barrier is impaired, such as severe malaria, treatments aimed at correcting volume deficits and improving tissue perfusion carry the risk of cerebral oedema [8,9]. We thus reasoned that volume resuscitation with colloids might be safer than crystalloidal solutions, as the latter freely equilibrate throughout the extracellular compartment and thus have the potential risk of accentuating intracranial pressure [3]. This hypothesis was tested in a previous phase II randomised controlled trial (see Text S1), which demonstrated that the resolution of acidosis was similar on either saline or albumin; however, a secondary analysis showed that albumin was associated with a significantly lower mortality (4%) than saline (18%), with the greatest benefit amongst those admitted in coma (5% versus 46%) [10].

Although it could be argued that further exploration of the optimal treatment regimes for patients with severe malaria acidosis should focus on albumin, there are reasons for caution in reaching this conclusion. The vast burden of childhood death from malaria is borne by the resource-poor countries of Africa, where provision of albumin as a resuscitation fluid is complicated by both cost and lack of availability. We therefore aimed to provide data on the safety and efficacy of a non-albumin colloid treatment for shock and correction of acidosis. A secondary objective was to establish whether a similar survival benefit could be achieved using a cheaper non-albumin colloidal solution to guide the selection of resuscitation fluids for inclusion in a phase III trial. Several types of colloid are available for clinical use—all differ in their molecular weight, colloid osmotic pressure, half-life in the intravascular compartment, and side-effect profile, mainly allergic reactions and adverse effects on coagulation [11,12]. We opted to test one of the modified gelatins, as these are the cheapest, most widely available colloid and have been shown in clinical trials to have a similar efficacy to albumin and other colloids [13–15]. Here we compare the safety and efficacy of Gelofusine (a succinylated modified fluid gelatin 4% intravenous infusion) and albumin in children with severe malaria complicated by metabolic acidosis.

## METHODS

### Participants

The study was conducted on the paediatric high dependency unit at the Kenya Medical Research Institute Centre for Geographic Medicine Research–Coast, Kilifi District Hospital, Kenya. Children greater than 3 mo of age presenting with either of the major clinical features of severe malaria—impaired consciousness (defined as either prostration or coma—Blantyre coma score [BCS] ≤ 2 [16]) or respiratory distress—were screened for inclusion in the study. Children were eligible for inclusion in the trial if they had all of the following criteria: Plasmodium falciparum parasitaemia, a clinical feature of severe malaria (as above), metabolic acidosis with a base deficit greater than 8 mmol/l, a haemoglobin of greater than 5 g/dl, and a clinical feature of shock [6]. Informed consent was obtained from all parents or guardians. Children with any of the following were excluded: pulmonary oedema (defined clinically as bilateral fine crepitations in association with sustained hypoxia [oxygen saturation < 95% measured by a pulse oximeter]), oedematous malnutrition, papilloedema, or refusal of consent. Ethical approval was granted to enrol children with clinical features of severe malaria who were critically ill on presentation (mainly decompensated shock), to start volume resuscitation without waiting for laboratory results.

### Interventions

Volumes administered in this trial were based upon earlier dose finding and safety studies [7]. In the earlier randomised controlled trial, volumes received were based upon the admission base deficit [10]: those with a base deficit of 8–15 mmol/l (moderate acidosis) received 20 ml/kg, and those with a base deficit of more than 15 mmol/l (severe acidosis) received 40 ml/kg. For the current trial we reasoned that to make patient care relatively independent of blood gas monitoring, hence the management protocol more generalisable to resource-poor settings, all children in this trial would receive an initial bolus of 20 ml/kg (a generic volume advocated by many paediatric protocols) except for children who presented with decompensated shock (systolic blood pressure < 80 mm Hg), who received 40 ml/kg over 1 h. A further bolus of 20 ml/kg was given to children 1 h later if they had persistent features of shock [17]. Shock was defined as not attaining all of the following resuscitation endpoints: heart rate and systolic blood pressure within threshold range for age, capillary refill time less than 3 s, and oxygen saturations greater than 95% in room air. Trial participants were continuously monitored for blood pressure, oxygen saturations, heart and respiratory rate, and electrocardiography using a Siemens (Munich, Germany) multi-channel recorder. Children in coma were also eligible for a double-blind placebo-controlled trial of fosphenytoin (the FOSCOM study) given at admission to prevent seizures. The primary endpoint of the FOSCOM study is post-recovery neurological sequelae. Projected completion date is the end of 2007. Otherwise, all other aspects of treatment were identical between the two groups. Our standard management has been reported previously [10]. Hyperkalaemia was treated with nebulised salbutamol and a bolus of glucose (2 ml/kg of 25% dextrose) after an initial cardioprotective dose of calcium gluconate (1 mmol/kg). Whole blood transfusion (20 ml/kg) given at any stage during admission was reserved for those whose haemoglobin fell below 4g/dl, or to less than 5g/dl if associated with respiratory distress. Ventilation facilities were not available, but children with short-term apnoea following convulsions were mask-and-bag ventilated.

On a case-by-case basis adverse events and deaths were reported to a data safety and monitoring committee and the Kenyan national ethics research committee. An interim summary of these events was provided in November 2005. The trial continued until completion, when 80 children fulfilling all admission criteria had been recruited. The trial received national ethical approval and was registered in September 2005 (ISRCTN 35536139) as one of two separate phase II trials; the second (assessing the safety of the rather more expensive colloids 6% dextran 70 and 6% hydroethyl starch) started in June 2006.

### Objectives

The study was designed to provide data on the safety and efficacy of Gelofusine infusion in children with severe malaria with respect to correction of shock and metabolic acidosis (primary endpoints) and adverse events including death (secondary endpoints). The overarching objective was to provide adequate information to help guide the selection of albumin, Gelofusine, or both for definitive multicentre trials. In order to assess whether the apparent survival benefit of albumin noted in earlier studies was solely due to colloidal oncotic properties or whether this effect could be achieved using a cheaper synthetic colloid, mortality was included as a secondary endpoint.

### Outcomes

Primary outcomes were resolution of shock (measured by those with shock at 1 h and 8 h) [17] and acidosis (percentage reduction of base deficit by 8 h). Secondary endpoints included in-hospital mortality, neurological sequelae at discharge, and other adverse events including potential complications of volume resuscitation (pulmonary oedema, raised intracranial pressure [defined as either a systolic blood pressure of more than the 90th centile for age in association with a falling heart rate, or papilloedema, or brain stem features of transtentorial herniation [18]], or allergic reaction). In phase II trials the use of mortality as an endpoint is not customary. However, our experience in two previous trials suggests that the surrogate endpoints (resolution of base deficit or shock [10] and seizure reduction [19]) did not predict significant differences in mortality. As the children eligible for this trial represent a high-risk group we therefore opted to include in-hospital mortality as a secondary outcome.

### Sample Size

Selection of candidate treatments for inclusion in costly and time-consuming phase III efficacy trials must inevitably be based on “an informed guess” as to the likelihood of any treatment proving to be beneficial in the proposed trial. This study was designed to provide sufficient safety data and some indication of the likely efficacy of volume expansion with a cheaper, more widely available colloid, Gelofusine, as compared with albumin, for which we already had considerable data to suggest a likely beneficial effect. The design of the current study and the numbers required to address these objectives were based upon the desire to provide sufficient data to inform our choice of fluids for inclusion in multicentre phase III trials, balanced by the necessity to minimise the exposure of children to a therapeutic intervention for which there is no available data in severe malaria. Formal sample sizes were therefore not calculated; we aimed to recruit 80 children: 40 to receive Gelofusine and 40 to receive albumin, to achieve our objectives.

### Assignment of Interventions

A quasi-randomised design was used, whereby fluid interventions were allocated sequentially in blocks of ten. In order to avoid bias due to user preference or patient selection, for ten consecutive patients only Gelofusine (Braun, Sheffield, United Kingdom) or 4.5% human albumin solution (Bio Products Laboratory, Elstree, United Kingdom) were available on the high dependency research unit for use in the clinical trial at one time, so the trial clinician had no discretion and also ensuring that cross-over was not possible. Otherwise the use of these fluids was strictly monitored, and they were not available for use outside of the clinical trial. Eligible children whose parents declined consent received saline boluses in accordance with local protocol.

### Blinding

Owing to the distinctive characteristics of the two study interventions, the need for rapid volume expansion, and the potential risk of dosing errors that may result if opaque administration sets were utilised, the intervention arms were not masked. Allocation of interventions was also not concealed.

### Statistical Methods

Analysis was performed using Stata version 8 (Stata Corporation, Texas, United States). The main analysis was by intention to treat (ITT). A secondary analysis examined only those complying with the trial protocol (per protocol [PP] analysis). Baseline and outcome variables were compared within each study arm using χ^2^ tests for categorical variables and ANOVA for continuous variables. The primary outcomes resolution of shock and acidosis were compared by χ^2^ tests and ANOVA, respectively. A dichotomous variable was created for shock for each of the time points 0, 1, and 8 h. A child was designated as in shock at each time point if he had either hypotension (systolic blood pressure < 80 mm Hg, or <70 mm Hg if <1 y) or two or more of the following: severe tachycardia (>180 beats per minute if <1 y, >160 if 1–6 y, and >140 if >7 y), delayed capillary refilling time (≥3 s), or oxygen saturations <95% [17]. The secondary endpoints death, neurological sequelae in survivors, and potential adverse effects of colloid resuscitation (pulmonary oedema, raised intracranial pressure, or allergic reaction) were compared between the intervention groups using a two-sided Fisher's exact test. In addition, we performed a sub-group analysis comparing mortality in the two intervention arms for cases presenting in coma (BCS ≤ 2), a group with an a priori increased risk of cerebral oedema, since this was the major rationale for using albumin. If the beneficial effect of albumin infusion went beyond its colloid oncotic properties and therefore reduced the risk of brain swelling, raised intracranial pressure, and ultimately death, then we would expect to see the greatest differences between the intervention arms in the coma sub-group. Unadjusted relative risk of death (and 95% confidence interval [CI]) was compared between albumin and Gelofusine.

As we have used identical criteria for patient enrolment, treatment, and assessment of outcome in this and previous phase 1 [7] and 2 [10] studies conducted at Kilifi, we performed a meta-analysis of the trials to provide a summary estimate of the effect on mortality of albumin. Data from the control group (that is, children not receiving volume resuscitation) of the phase II trial were not included in this analysis, on the grounds that the control group was not comparable in terms of a priori risk of fatal outcome, since the control group included only a relatively low-risk group with base deficit 8–15 mmol/l (moderate acidosis), whereas those receiving saline or albumin also included a high-risk group with base deficit above 15 mmol/l (severe acidosis). We used summary data for each arm of the trials and employed standard methodology and the Stata add-in command “metan” to calculate relative risk [20].

## RESULTS

### Participant Flow

**Figure 1:**
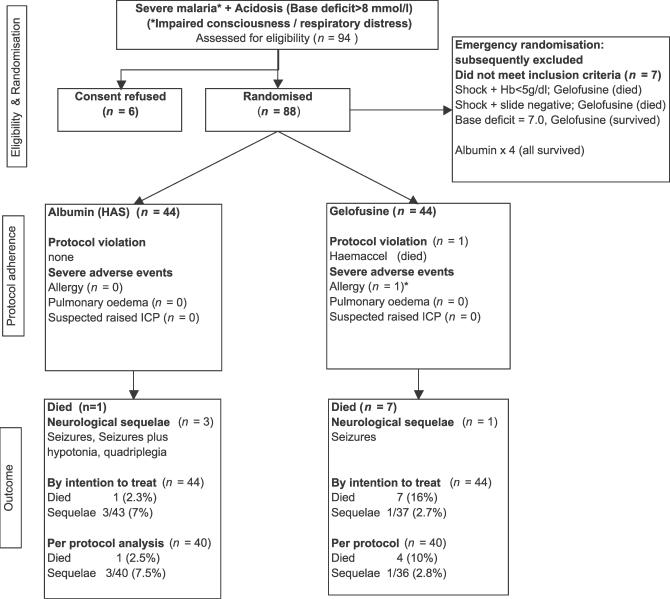
Trial Flow HAS, human albumin solution; ICP, intracranial pressure.

During the study period 94 children were clinically eligible for trial recruitment; in six cases parental consent was not granted. Seven cases were assigned as emergencies to receive study interventions, but were subsequently found not to have met with all the inclusion criteria (see Figure 1). There were no protocol deviations and only one protocol violation: a child allocated to Gelofusine received Haemaccel (another gelatin-based colloid) in error. The PP analysis includes only those fulfilling all inclusion criteria and adhering to trial protocol. Compliance with trial protocol was high, and there were no withdrawals from the study and no losses to follow-up for in-hospital primary or secondary outcome.

### Recruitment

Enrolment began in December 2004 and ended in January 2006, when the projected study sample size had been accomplished.

### Baseline Data

Admission characteristics are summarised in Table 1. Albumin and Gelofusine groups were well matched with regard to baseline clinical and biochemical markers of severity. Up to 60% of the trial participants were in coma (BCS ≤ 2) at admission, an a priori risk factor for poor prognosis. Although pre-selected by eligibility criteria (impaired consciousness or respiratory distress and acidosis), all children had one or more additional feature of hypovolaemia including severe tachycardia (heart rate > 160 beats per minute), delayed capillary refill time (≥3 s), hypoxia (oxygen saturations < 95% on pulse oximetry), or hypotension. Moreover, most fulfilled a more stringent international definition of shock (hypotension or two of the three features defining shock). The majority had decompensated metabolic acidosis with low pH, low bicarbonate, increased base deficit, and/or a high lactate.

**Table 1:**
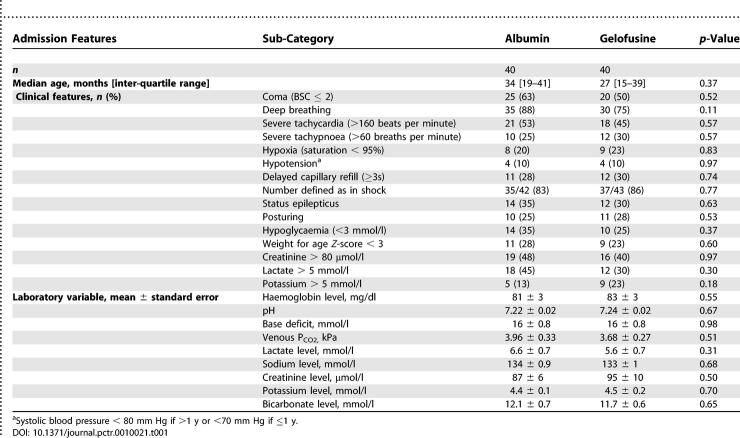
Baseline Characteristics

### Numbers Analysed

A total of 88 children were included in the ITT analysis (*n =* 44 for albumin and *n =* 44 for Gelofusine) for primary and secondary outcomes, including the seven children allocated to interventions under the emergency consent waiver (see Figure 1). Eighty children were included in the PP analysis (*n =* 40 for albumin and *n =* 40 for Gelofusine) that excluded the seven cases presenting as emergencies (as they did not fulfil all inclusion criteria) and the child who received Haemaccel rather than Gelofusine.

### Outcomes and Estimation

#### Primary outcomes.

Overall, the resolution of shock and metabolic acidosis were similar between the groups (Table 2). By 4 h, all trial participants had attained the level one goals for resuscitation (data not shown); most (56/78; 72%) had either no features of shock or only one of the four features required for further intervention and thus no further fluid boluses were given after this point. Coma resolution, defined by the ability to localise a painful stimulus (equivalent to BCS > 2), occurred more gradually over the first 24 h and was comparable between the groups. By 24 h, 10/39 (26%) of the albumin group and 7/37 (19%) of the Gelofusine group remained in coma (*p =* 0.59).

**Table 2:**
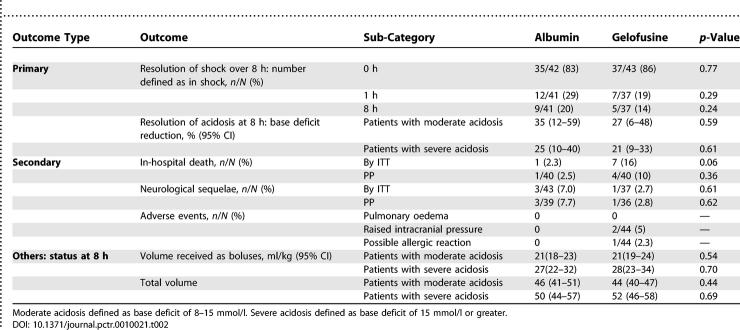
Major Outcomes

#### Secondary outcomes.

By ITT analysis, death occurred in 1/44 (2.3%) of the albumin-treated patients compared to 7/44 (15.9%) of the Gelofusine-treated patients (Fisher's exact test, *p* = 0.06). Considering only those patients complying with the protocol, fatal outcome occurred in 1/40 (2.5%) of albumin-treated children and 4/40 (10%) cases receiving Gelofusine (*p =* 0.36). In the sub-group admitted in coma, mortality in the albumin arm was 1/25 (4%) for both ITT and PP analysis and 6/23 (26%; *p =* 0.04) and 3/20 (15%; *p =* 0.31) for ITT and PP analysis, respectively, in the Gelofusine arm. Neurological sequelae developed in 3/43 survivors receiving albumin (by ITT, 7.0%) and in 1/37 survivors receiving Gelofusine (by ITT, 2.7%; Fisher's exact test, *p =* 0.62), or 3/39 (7.7%) and 1/36 (2.8%; *p =* 0.61), respectively, treated PP (see Table 2). All four cases that developed neurological sequelae presented in coma at admission; three cases had evidence of antecedent neurological impairment (Table 3). Those with pre-morbid epilepsy were reported to have increased seizure frequency and severity 1 mo after discharge.

**Table 3:**
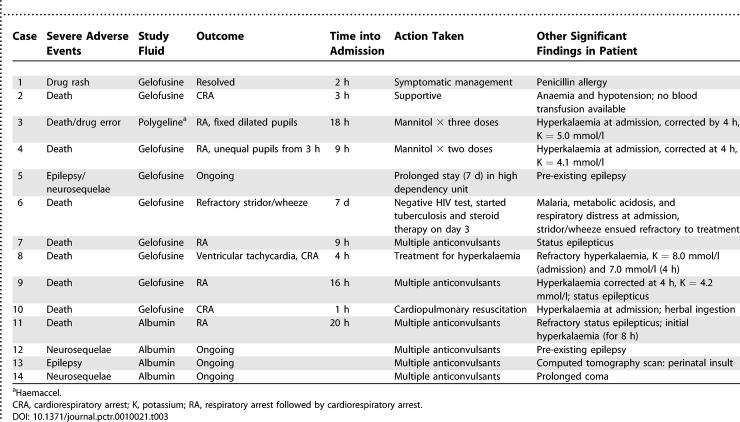
Summary Data on Severe Adverse Events

There was no evidence of pulmonary oedema or fluid overload in any trial participant; however, fatal neurological events were more common in the group receiving gelatin-based intervention fluids (Table 2). Brain swelling and transtentorial herniation were suspected in two children assigned to Gelofusine (cases 3 and 4; Table 3). Unilateral pupillary dilatation was noted at 4 h and 3 h into admission for cases 3 and 4, respectively; both patients went on to develop bilateral fixed dilated pupils before having a terminal respiratory arrest, despite an infusion of mannitol. Two further deaths in the Gelofusine group (cases 7 and 9) in addition to the sole albumin fatality (case 11) were associated with refractory status epilepticus, where optimal seizure control was compromised by respiratory depression. The other fatalities were complicated by anaemia and hyperkalaemia (two recognised complications of severe malaria) and wheeze/stridor. For cases complicated by hyperkalaemia, continuous electrocardiography monitoring did not detect obvious arrhythmias, and potassium levels fell into the normal range (see Table 3) except for one case, which terminated in ventricular tachycardia. Allergic reaction to Gelofusine was suspected in one case; however, the generalised red rash (without adverse cardiovascular effects) occurred immediately after the initial dose of intravenous benzylpenicillin, and over 1 h after the bolus of Gelofusine.

### Ancillary Analyses

**Figure 2:**
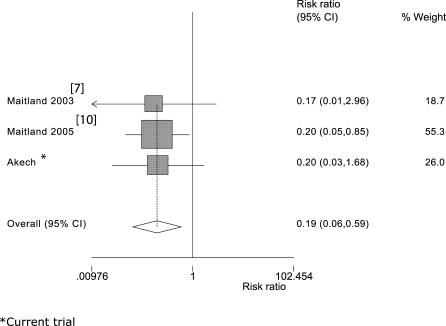
Summary Estimate of the Effect of Albumin on Mortality A meta-analysis of published trials [7,10] and the current trial comparing the relative risk of death in children with malarial acidosis managed with albumin bolus versus other fluid boluses (saline or Gelofusine).

A PubMed search using the terms “malaria” and “child” and “hypovolaemia” and “therapy” found that the two previous Kilifi-based trials [7,10] and the current trial were the only ones appropriate to include in a meta-analysis. A further trial was identified; however, this was conducted in children with severe anaemia so was not relevant to this meta-analysis [21]. The meta-analysis showed a pooled relative risk of death with albumin administration of 0.19 (*p =* 0.004; 95%CI 0.06–0.59) compared to saline or Gelofusine fluid boluses (Figure 2). These trials included 238 eligible children, of whom 112 were treated with albumin, 86 treated with saline, and 40 treated with Gelofusine [10]. These three trials have shown a consistent survival benefit in patients receiving albumin (Table 4); mortality from the combined data for 112 albumin-treated patients was only 2.6%. This low mortality on albumin treatment is striking when contrasted with previously published mortality rates, and with current unpublished information from other centres (Table 4). Mortality rates for saline-treated patients (18/%) and Gelofusine-treated patients (16%) are similar to these previous reports (Table 4). Nevertheless, whilst most of the children included in these case series did not receive fluid boluses, a large number may have received a whole blood transfusion (a form of volume expansion) because of the unscheduled use of transfusion (for children with haemoglobin > 5 g/dl), which may in part account for the lower mortality at these sites.

**Table 4:**
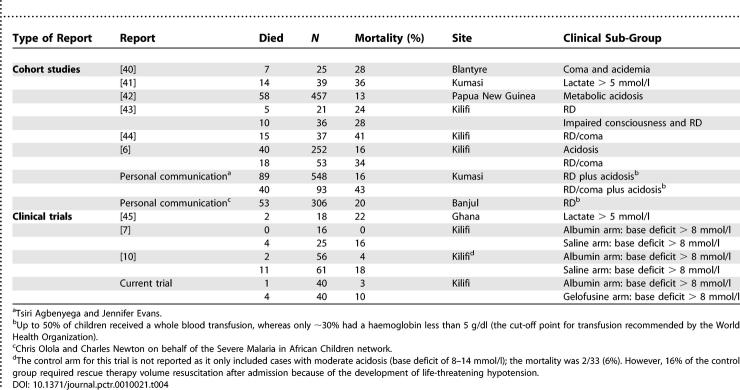
Global Summaries of Mortality in Children with Severe Malaria and Acidosis

## DISCUSSION

### Interpretation

These results augment the conclusions of our previous trials, designed to establish whether hypovolaemia was an important complication of severe malaria in African children, to examine whether volume resuscitation represented a safe and effective therapeutic intervention [7,10,21], and to establish whether this could be more safely achieved with albumin or other colloids than crystalloid. Both Gelofusine and albumin were found to be equally effective in treating shock and correcting acidosis uncomplicated by pulmonary oedema or fluid overload, but fatal neurological complications were greater in children receiving gelatin-based infusion (4/44; 9%) than in those receiving albumin (1/44; 2.4%). Even though the current study was not powered to detect differences in mortality, our findings in this trial, like those of our previous phase I and II studies, suggest that albumin represents a superior resuscitation fluid in terms of survival, particularly in cases complicated by coma—a group we hypothesized would receive the greatest benefit. Mortality in the coma sub-group receiving Gelofusine (6/23; 26%) was substantially greater than in those receiving albumin (1/25; 4%). In all three trials, we were unable to identify a clinical surrogate marker for outcome, suggesting that further physiological studies are needed to elucidate the precise mechanism of protection. Neurological sequelae were more common in survivors receiving albumin in this and our previous trial [10], but their frequency accords with all other case series of severe malaria known to us, which have consistently documented ~12% neurological impairment rates in survivors [16,22–25]. Small trials are susceptible to imbalance in admission characteristics, prognostic markers, and, in trials of volume expansion, volumes administered. These random fluctuations may be further exaggerated in an open, non-randomised trial without concealment of participant allocation to interventions. The albumin and Gelofusine groups were well matched in terms of baseline and prognostic variables and volumes received. However, the primary outcome was resolution of shock, assessed clinically, which may be influenced by clinician bias or affected by the administration of equal volumes of intervention fluids whose colloid oncotic properties may not be equivalent in terms of volume expansion. Nevertheless, we found that most fatalities in the Gelofusine arm were neurological in origin, rather than a result of cardiovascular collapse, which would be more usual if shock were inadequately corrected. Despite the limitations of the trial design, our main finding of a difference in mortality, for which the major prognostic factor at admission is coma, is less likely to be affected by our trial design. Indeed, coma was more common in the albumin group at admission, which is worthy of consideration when interpreting the study findings.

### Generalisability

Administration of fluid boluses in this trial was based on bedside assessments of shock that tested the generalisability of the treatment protocol—since blood gas analysis is not widely available in Africa. In this regard the protocol was successful and resulted in a similar mortality benefit in the albumin arm to that shown in our previous randomised controlled trial. The striking effect of albumin on mortality is further evidenced when compared to reports from equivalent case series. These have consistently documented mortality rates of 16%–43%, with the greatest mortality occurring in groups where acidosis or its clinical correlate, deep breathing, is complicated by coma (Table 3). The beneficial effect of albumin infusion, beyond that offered by Gelofusine, points to specific neuroprotective effects that act through additional mechanisms other than improving colloid osmotic pressure. Albumin has a range of physiological effects in addition to those operating via its colloidal properties [26], which may result in the amelioration of brain swelling [27,28] and the improvement of blood flow to critically perfused brain regions [29]. Albumin exerts direct effects on vascular endothelium, by binding to the endothelial glycocalyx to maintain normal permeability [30], and exerts complex influences on erythrocyte aggregation by increasing low-shear viscosity but decreasing erythrocyte sedimentation under no-flow conditions [31,32]. These non-colloid properties of albumin are increasingly recognised to be of therapeutic importance in the treatment of stroke [33] and may be of particular importance in the pathophysiology of cerebral malaria, where adherence of parasitised red blood cells to the endothelium, aggregation of red cells due to the phenomenon of rosetting, and impaired red cell deformability [34] may be influenced by the highly negative charge of the albumin molecule [26].

### Overall Evidence

The uncertainty surrounding the use of resuscitation fluids in children presenting with acute falciparum malaria is of critical importance from the perspective of health professionals who are responsible for the emergency management of children in endemic areas, and needs to be definitively addressed in large multicentre trials [35]. The debate over the merits of volume resuscitation using colloids or crystalloids in hypovolaemic shock often fails to recognise that distinct pathophysiological mechanisms are involved in shock in patients with different underlying diseases, and in children as compared with adults. Large comparative studies (for example, [36]) and systematic reviews of them [37] have failed to identify clear benefits of resuscitation with albumin over resuscitation with crystalloids in heterogeneous populations of adults with shock complicated by a wide range of underlying disorders. However, we believe this should not preclude consideration of albumin in paediatric trials, particularly in one involving a single disease entity with a unique pathophysiology. Although severe malaria has many features in common with severe sepsis, and hypovolaemia may be an important contributor to the pathophysiology of both conditions, there are unique pathophysiological processes involved in severe malaria that are clearly distinct from those occurring in sepsis and in other causes of hypovolaemic shock. Hypovolaemia, even of only modest degree, would augment processes, including cytoadhesion of parasitised red blood cells to vascular endothelium, rosetting, and reduced red blood cell deformability [2], that lead to a degree of microvascular obstruction and further compromise organ perfusion. Albumin may act to improve microvascular perfusion in malaria through its rheological effects as well as by volume expansion and influencing fluid shifts across the endothelium.

The three studies we have undertaken, including a meta-analysis, point to a dramatic effect of albumin on the outcome of severe malaria in children, and a clear benefit as compared with other fluids, and suggest that further evaluation of albumin in children with severe malaria, in large phase III studies, is now needed. The comparable mortality of Gelofusine treatment with that of saline observed in our previous studies suggests that inclusion of an additional synthetic colloid arm is unwarranted, as the increase trial size necessitated by a four-arm trial (albumin, saline, Gelofusine, and maintenance-only control) is not justified by the small possibility that Gelofusine may be preferable to saline.

In addition to efficacy and safety, cost and availability of fluids are important issues. While the roll out of costly antiretroviral drugs in sub-Saharan Africa has been justified on the basis of the scale of the HIV epidemic, any decision to make albumin widely available would also require an in-depth cost-effectiveness analysis. Setting aside the possible effects of albumin on decreasing the length of hospital stays and reducing the costs and risks to patients from transfusions, and its potential effect on neurological sequelae, a “back of the envelope” calculation suggests that albumin would be highly cost effective, at around US$30–50 per life saved at current prices. Relevant, but often overlooked, is the cost of a blood transfusion, estimated at US$30 in most localities, which is very commonly and inappropriately prescribed in such cases, despite concerns about its microbiological safety [38]. Furthermore, if albumin were shown in future trials to result in significant benefit, then most African countries already have the infrastructure within large regional transfusion units to produce a microbiologically safe albumin at low cost if fairly simple and robust technologies are introduced [39]. Paradoxically, albumin, rather than other synthetic colloids, would then represent the cheapest and most readily available colloid. What is currently missing is the scientific evidence and imperative to justify introduction of these technologies. Moreover, improving childhood survival through the application of evidence-based simple therapies such as volume resuscitation will have benefits beyond the boundaries of severe malaria because of improvements in childhood survival.

## SUPPORTING INFORMATION

CONSORT Checklist Click here for additional data file. (42 KB DOC)

Trial Protocol Click here for additional data file. (150 KB DOC)

Text S1 Appendix (21 KB DOC)

## Abbreviations

BCS: Blantyre coma score
CI: confidence interval
ITT: intention to treat
PP: per protocol
