# Correction: Volume Expansion with Albumin Compared to Gelofusine in Children with Severe Malaria: Results of a Controlled Trial

**DOI:** 10.1371/journal.pctr.0010037

**Published:** 2006-11-24

**Authors:** Samuel Akech, Samson Gwer, Richard Idro, Greg Fegan, Alice C Eziefula, Charles R. J. C Newton, Michael Levin, Kathryn Maitland

In *PLoS Clinical Trials*, volume 1, issue 5: DOI: 10.1371/journal.pctr.0010021

An error was made in the editorial commentary. The correct text should read as below.

**What this trial shows:** The investigators found no significant differences in the primary outcomes (correction of shock and acidosis in the blood 8 h after fluids were started) or in death rates between children given Gelofusine and those given albumin. The researchers then combined the data on death rates from this trial with data from two other trials with an albumin arm. This combined analysis suggested that death rates with albumin were lower than with other fluids, either Gelofusine or salt solution.

