# Supplementary material for: Volume Expansion with Albumin Compared to Gelofusine in Children with Severe Malaria: Results of a Controlled Trial
Source: PLoS Clin Trials. 2006 Sep 15;1(5):e21. doi: 10.1371/journal.pctr.0010021 (PMC1569382; doi:10.1371/journal.pctr.0010021)
Supplement: Trial Protocol [file pctr.0010021.sd002.doc]

# **1. TITLE**

# **Safety and dose finding study of a modified gelatin colloid (Gelofusin®) for correction of volume deficits in severe malaria**

**2. INVESTIGATORS AND INSTITUTIONAL AFFILIATIONS**

Dr. Samuel Akech^1^

Dr. Samson Gwer^1^

Dr. Richard Idro^1^

Dr. Greg Fegan^1,2^

Dr. Chi Eziefula^1^

Prof. Charles RJC Newton^1,3^

Prof. Michael Levin^4^

Dr. Kathryn Maitland^1,4^ –Principal Investigator

1. Centre for Geographic Medicine Research (coast), Kenya Medical Research Institute, PO Box 230, Kilifi, Kenya
2. Infectious Diseases Epidemiology Unit, London School of Hygiene and Tropical Medicine, London, WC1E 7HT, UK
3. Neurosciences Unit, Institute of Child Health, The Wolfson Centre, Mecklenburgh Square, London, WC1N 2AP, UK
4. Department of Paediatrics and Wellcome Trust Centre for Clinical Tropical Medicine, Faculty of Medicine, Imperial College, Norfolk Place, London, W2 1PG, UK

## 3. SUMMARY

Over the past few years we have conducted a series of studies that have provided new, clear evidence for the presence of hypovolaemia in severe malaria. These were the subject of investigation in a previous proposal: ‘The role of hypovolaemia in the acidosis of severe malaria in children’ (SCC no 603). We demonstrated that volume expansion with either normal saline or 4.5% human serum albumin corrected the haemodynamic abnormalities and was associated with improved organ function and reduction in acidosis. In a formal randomised controlled trial we showed that volume expansion with albumin was associated with a significantly lower mortality in children with severe malaria acidosis, especially those admitted in coma. As human albumin solution is costly and not available in Africa in this current study we aim to examine the safety and dose required (efficacy) for the correction of hypovolaemia of a lower cost colloid, Gelofusin in children with severe falciparum malaria complicated by acidosis. This will be compared to a ‘control’ group of children receiving albumin. In this prospective study we aim to enrol 80 children and randomised them to either Gelofusin or albumin. The primary endpoints will be to compare the safety and efficacy of both colloids on the resolution of haemodynamic markers of shock. Secondary end points will be adverse events, including alterations in coagulation homeostasis. The results of this study will form the basis for the future design of multicentre trials comparing colloidal solutions with saline or maintenance alone, which are required before specific treatment recommendations can be made.

## 4. BACKGROUND

In severe malaria, acidosis has been shown to be the best independent predictor of death^1^. Acidosis in most critically ill children, regardless of the underlying causative disease, is most frequently associated with impaired perfusion, and is generally, treated by volume expansion, optimization of tissue and organ perfusion and the provision of adequate glucose to reduce metabolic production of organic acids^2^. However, in severe malaria, although current recommendations include volume expansion^3^, there has been concern that some patients are at risk of developing cerebral or pulmonary oedema, which has resulted in most patients receiving maintenance fluids only or modest volume restriction. Both these complications are infrequent in African children with severe malaria^3^. Similar fears were raised in the past when treating children with septicaemia complicated by severe central nervous system infection. Subsequently, it has been recognised that fluid restriction is detrimental to brain perfusion in this situation^4,5^. Moreover, recent studies investigating the role of aggressive early fluid resuscitation in paediatric septic shock (defined by acidosis)^6^ and dengue shock syndrome^7^ have demonstrated significant improvements on survival. In neither study were there increases in the rate of pulmonary oedema or clinical features of cerebral herniation following fluid resuscitation, lending crucial support to the premise that volume expansion is safe in a number of severe infections, regardless of aetiology.

**Evidence for hypovolaemia in severe malaria**

## During the past few years we have conducted a series of studies that have demonstrated new, clear evidence for the presence of hypovolaemia in severe malaria. These were covered by a previous proposal: ‘The role of hypovolaemia in the acidosis of severe malaria in children’ (SCC no 603). In summary;

## Retrospective review: In this case note review we found that many of the clinical and laboratory features associated with a poor outcome are recognizable consequences of impaired tissue perfusion; including acidosis (base excess <-8 and/or its clinical correlate, deep breathing), hypotension, dehydration and elevated creatinine. Ancillary features, regarded as part of the clinical picture of the sepsis syndrome, including hypoxia, hypoglycaemia and electrolyte disturbances, were also important factors in predicting a fatal outcome. Overall, shock (hypotension or two or more other features) was present in 84% of deaths. Elevated creatinine, biochemical or clinical features of acidosis and delayed capillary refill time (≥3s) were numerically the three major contributors to the shock score of ≥2^8^.

## Pilot studies: We conducted a prospective phase I study examining the safety of volume expansion for children admitted with clinical features of severe malaria (impaired consciousness or deep breathing) complicated by acidosis (base excess <-8). Patients recruited to the study underwent continuous monitoring of blood pressure, pulse rate, respiratory rate, oxygen saturation and central venous pressure using a multichannel recorder. Fifty-three children were recruited and all had evidence of compensated shock on admission, with tachycardia, tachypnoea and prolonged capillary refill time (≥3s). Mean central venous pressure (CVP) [standard error] at admission was low: 2.9 [0.5] cm water (see Figure); in the group with severe acidosis (base excess<-15) 44% had hypotension (systolic blood pressure < 80 mmHg). Improvements of haemodynamic indices and a reduction in acidosis followed administration of either saline or albumin. By 8 hours the mean CVP had increased to 7.5 [0.5] cm water (F=34.4, P<.001) and was associated with a reduction in mean respiratory rate 49 [2] to 41 [1], F=7.0, P=.009, reduced tachycardia 151 [5] to 141 [3], F=3.4; P=.06 and reduced capillary refill time. No child developed evidence of the life threatening complications of pulmonary oedema and raised intracranial pressure. We concluded that both albumin and saline were safe and that doses of between 20-40mls/kg given over the first hour after admission resulted in a correction of the features of hypovolaemia and resolution of the acidosis^9^.

Figure: Central Venous Pressure measurement in children with malarial acidosis: correction of hypovolaemia with volume replacement

Central venous pressure (CVP) measures the pressure in the column of blood returning to the heart (preload). In health, this is normally 5-8cm H_2_O. In sick children higher preloads ~ 8-10cm are often required. In these children with severe malaria complicated by acidosis CVP was low at admission (0 hours) and normalised following 20-40mls/kg intravenous bolus of 0.9% saline or 4.5% albumin (indicated by the diamond).

## Phase II randomised controlled trial:

## Aims: We aimed to test the hypothesis that volume expansion with a colloidal solution would enable the acidosis and hypovolaemia of severely ill children with malaria to be corrected with less risk of precipitating cerebral oedema, than if crystalloid was used. In this trial we chose to use 4.5 % human albumin solution for the colloid and 0.9% saline as the crystalloid^10^. The use of albumin as a major resuscitation fluid for septic shock is well established in European and Australian paediatric critical care and has been associated with a general reduction in mortality with the least adverse reactions^11,12^, despite the results of a recent meta-analysis^13^.

## Design and results: We randomly assigned children with severe malaria and metabolic acidosis (base excess <-8) to treatment with either albumin or saline: patients with moderate acidosis (base excess between –8 and -15) received 20 ml/kg; and those with severe acidosis (base excess <-15) received 40 ml/kg. For ethical reasons, a control (maintenance only) group was only included for patients with moderate acidosis. 150 children were enrolled. There was no difference in the rate of resolution of acidosis between the groups; however, requirement for alternative treatments was significantly more common in the maintenance only control group (P =0.004). Overall, mortality was significantly lower in patients receiving albumin (2/56; 3.6%) than those treated with saline (11/61; 18%) (χ^2^= 6.1, P=0.01). The improved mortality was entirely confined to the children admitted in deep coma, where mortality was (1/21; 5%) in albumin recipients as compared with (11/24; 46%) in saline recipients (P =0.002). Only one child developed pulmonary oedema (in the saline group) (overall incidence <1%) and 10 (7%) of the 135 survivors had neurological sequelae. Prior to the study mortality in children with malaria and severe acidosis had been consistently documented at between 25-40%, hence the low rate in the albumin recipients (4%) is lower than any previously reported patients with similar severity of disease. Our finding that the beneficial effect of albumin was greatest in the comatose patients (5% in the albumin treated group) and that most of the deaths in the saline treated patients appeared to be neurological in origin, is consistent with the predicted physiological effects of albumin^14^.

## Interpretation of the findings

Treatments aimed at correcting volume deficits and improving tissue perfusion carry the risk of increasing cerebral oedema. Non-colloidal containing solutions such as normal saline are freely distributed across the extracellular compartment and will move rapidly from the intravascular compartment into the tissues with the potential risk of accentuating RICP and causing pulmonary oedema. This risk is greatly increased by the presence of hypo-albuminaemia. Children with severe malaria frequently have hypo-albuminaemia, a factor we reckoned that would favour the use of colloid replacement^10^. In this study we demonstrated some support for this principle, since the major difference in mortality between saline and albumin treated children was limited to the high-risk group presenting with respiratory distress and coma. A number of additional questions arise from the findings of this trial.

First, maintenance only fluid was only given to the lower mortality group with moderate acidosis, in the absence of a control group receiving maintenance fluids alone in those with severe acidosis, where most deaths occurred, it is not clear whether saline administration is simply inferior to albumin, but preferable to fluid restriction, or whether saline administration is actually hazardous. Second, since saline is the standard resuscitation fluid in use across Africa, and albumin is expensive and currently unavailable in most African centres, further work is required before definitive treatment recommendations can be made. Finally, now we demonstrated the principal that volume resuscitation with albumin maybe superior to saline in those with coma the safety profile of alternative cheaper colloid should be examined. In this proposal we aim to examine the safety and efficacy of the gelatin-based colloids in the treatment of acidosis of severe malaria.

## Choice of colloids

There are 4 general types of colloid products available for clinical use; albumin, dextrans, starches and gelatins^15^. They differ in their molecular weight, colloid osmotic pressure and duration within the intravascular compartment. Albumin, prepared from pooled human donors, has the best safety profile but is in short supply and is therefore expensive.

**Dextrans**: Dextrans (Dextran 40 and Dextran 70) are glucose polymers that initially produce intravascular volume expansion by increasing osmotic load, but their effect is transient. Smaller polymers (Dextran 40) rapidly escape from the intravascular compartment; 50% of the dextran polymer is lost within 3 hours, whilst the larger polymers within Dextran 70 escape less rapidly (35% clearance within 12 hours)^16^. The main route of excretion is renal, some of the extravascated dextran remains in the

Table one: a summary of the characteristics and properties of the colloids commonly used in critical care.

|  |  | Dextrans | | Starches | | Gelatins |
| --- | --- | --- | --- | --- | --- | --- |
|  | **Albumin** | **Dextran 40** | **Dextran 70** | **Hetastarch (6%)** | **Pentastarch** | **Gelofusin** |
| Molecular weight (KD) | 70 | 40 | 70 | 450 | 260 | 30 |
| Plasma volume expansion (ml/500ml infused) | 500 | 500-1000 | 500-700 | 500-700 | 600-800 | 500 |
| Cost (US $) per litre (in USA) | 360 | 200 | 150 | 130 | 150 | 15-20* |
| Incidence of allergic reactions | 0.011 | 1.1 | 1.1 | 0.09 | Not available | 0.06 |
| Effect on clotting | v. mild, dilutional | Decreases in von Willebrands factor |  | Increase in prothrombin and partial thromoboplastin times |  | v. mild, dilutional |
| Comment |  | Decreases blood viscosity | Increases blood viscosity | Prolongs bleeding time |  |  |

* not available USA, UK equivalent price given

extravascular space and exert an osmotic effect. Another major feature of the dextrans is that they lower blood viscosity and thus are favoured for use during surgery, as they decrease the risk of deep vein thrombosis and pulmonary embolus^17^. Dextran administration is associated with a coagualopathy (decreased fibin clot formation), decreased levels of von Willebrand factor (vWF) and associated factor VIII:c activity. The decrease in wVF and VIII:c are greater than expected by dilution. VWF is the ligand between the platelets surface and the endothelial wall and this has resulted in prolonged bleeding times in vivo^18^. The rate of allergic reaction is 1.1%.

**Starches:** Hetastarch (hydroxyethyl starch, HES) is produced by partial hydrolysis of amylopectin, which is made more soluble by the addition of glucose molecules. The 6% solution is the most commonly used solution (Hepaspan) and closely resembles 5% albumin; both are isotonic and increase the intravascular volume by the amount infused. The half life of hetastarch is longer (24 –36hours) than albumin^15^. It is both excreted by the kidneys and degraded by the enzymatic action of amylase. The major set back is that it alters haemostasis leading to increases in prothrombin time, partial thromboplastin time and bleeding time, and decreased levels of VIII:C and VIII:Ag^18^. As a result, the maximum daily dose recommended for adults is 1500mls/day and for children is 20mls/kg^15^. Pentastarch has a greater osmotic pressure and thus leads to greater volume expansion, nearly twice that of the volume infused. However, the half life is only 2.5 hours; 60% is excreted in the urine. Furthermore, Pentastarch is associated with similar haemostatic problems as Hetastarch. Allergic reactions are rare; < 0.1%.

**Gelatins:** The gelatins are produced from alkali-treated bovine collagen, which hydrolyses the esters and peptide links, followed by suspension in aqueous solution. There are two types of gelatins the succinylated gelatins (Gelofusin) or urea-linked gelatins (Haemacell). Both types of solutions are polydisperse. The small particles exert a large osmotic effect, but are then rapidly lost by glomerular filtration^15,17^. The volume expanding effect last for ~4 hours^17^. Compared to human albumin, the modified gelatins have similar shown efficacy in formal randomised trials^19^. Reports of haemostatic abnormalities after infusion of gelatins are rare and in vitro changes are related to dilution rather than a direct effect on the clotting cascade^18^. Allergic reactions are also rare < 0.1%. Haemacell has a higher calcium (6.26mmol/L) (which may lead to clotting if infused with blood) and higher potassium (5.1mmol/L) than Gelofusin^15^. Thus, the modified gelatins, in particular Gelofusin, emerge as the colloid with, theoretically, the least anticipated complication rate and for this study will be considered in this current study as the colloid of choice.

**Theoretical considerations for the use of colloid in severe malaria**

Most of in-vivo trials examining the effect of different colloids on haemostasis (usually measure by blood loss) have been conducted in clinically stable patient groups undergoing corrective orthopaedic or cardiac surgery. In septic shock coagulation and haemostasis is disordered. In severe malaria bleeding secondary to disseminated intravasular coagulation (DIC)^3^ and procoagulant activity has been reported in adults with severe malaria^20,21^. In children whilst DIC is rarely encountered, little to no information exists on the abnormalities in coagulation and homeostasis^22^. There are however, a number of considerations when considering the use of non-albumin colloids. Firstly, in severe malaria moderate degree of thrombocytopenia is invariable^22^. In children admitted with severe malaria to KDH, thrombocytopenia (platelets counts < 150x10^12^) is common (>55%)^23^. Dextrans and starches are known to reduce von Willebrand factor and increase the risk of bleeding^18^. In the presence of thrombocytopenia, a von Willebrand-like syndrome is a possible complication. Albumin has no effect on coagulation and the effects by gelatins are mild, usually related to a dilutional effect^18^.

**5. JUSTIFICATION**

We have previously showed that rapid volume expansion with 4.5% albumin shortly after admission is safe and has lead to a significantly lower mortality in children with severe malaria acidosis, especially those admitted in coma. As human albumin solution is costly (£45 per 500mls in the UK ~KSH 5,850) and not easily available in Africa we aim to examine the safety of a lower cost licensed colloid, Gelofusin (£5 per 500mls ~KSH 650) when used to correct hypovolaemia. The safety and dose response will be compared to that of albumin. Since most non-albumin colloids have some effect upon coagulation, part of this trial will include serial measures of coagulation activation[[1]](#footnote-2).

**6. NULL HYPOTHESES.**

1. There is no difference in the safety profile or effect on physiological parameters of shock using gelatin-based colloids and albumin for the correction hypovolaemia in children with severe malaria.
2. The use of either of the two colloids will lead to no changes in coagulation after administration.

##### 7. OBJECTIVES

**Primary Aim**

1. To establish, through prospective intervention studies, whether volume depletion (hypovolaemia) and acidosis complicating severe malaria can be safely corrected by volume replacement with Gelofusin.

### Secondary aims

1. To establish whether volume correction by Gelofusin can be undertaken safely and is comparable to albumin.
2. To examine whether the volume of Gelofusin required for correction of the physiological parameters of hypovolaemic shock is similar to albumin.
3. To establish whether albumin or Gelofusin lead to derangements of coagulation.

##### 8. DESIGN AND METHODOLOGY

**a) Study Site.**

The study is to be based on the high dependency unit at the Centre for Geographical Medicine (Coast) and Wellcome Trust Laboratories at Kilifi District Hospital (KDH), Kenya.

**b) Study Population**

Children > 3 months old with severe falciparum malaria will be considered.

## Inclusion criteria

Presence of a severity feature (impaired consciousness and/or deep breathing) plus the presence of metabolic acidosis (base excess <-8).

## Exclusion criteria

Children of families who decline consent will receive standard management only. Children with severe malaria anaemia (haemoglobin < 5g/dl). Children with CSF changes consistent with meningitis. Children with clinical features of pulmonary oedema (defined as clinical evidence presence of fine crepitations in both lungs plus oxygen saturations < 95%). Those with evidence of raised intracranial pressure (brain stem features of coning, systolic blood pressure >90% centile for age plus falling heart rate and/or papilloedema) or any conditions that may contraindicate the use of volume replacement e.g.: established renal failure or known congenital heart disease.

##### c. Sampling

1. **Sample size determination**

These are pilot studies designed primarily to examine the safety of Gelofusin in children with severe malaria complicated by acidosis. As Phase I studies they are designed to provide adequate data to justify progression to next phase ie: multicentre studies, but with the desire to avoid exposure of a large group of children to a therapeutic intervention for which there is no available data in severe malaria. The trial will therefore, provide important data on the volumes required of Gelofusin in achieving satisfactory improvements in haemodynamic features of shock such in a similar manner to that which has been previously observed in children given albumin^9^. Formal sample sizes will therefore not be calculated. We aim to recruit 80 children: 40 will have moderate acidosis and 40 will have severe acidosis. 20 children in each group will receive Gelofusin and 20 will receive albumin. Since we showed that the greatest benefit of albumin was in those with coma^24^, at least 50% of the children recruited will have coma. Gelofusin, would be used in preference to albumin in larger studies if mortality were 10% (confidence interval 7.5-12.5%) or less.

**ii. Sampling procedure**

Eligible children will be randomised to receive either Gelofusin or 4.5% human albumin solution. Most children will receive 20-40mls over the first hour, dependant upon degree of acidosis and physiological improvements in shock. Otherwise, the management the children receive will be identical to our current standard of care. Two groups will be considered:

**Group A: Moderate acidosis**

Children will receive 20mls/kg of either Gelofusin or 4.5% human albumin solution. This will be given over the 30minutes following admission. However, the final volumes given will depend upon the attainment of pre-defined resuscitation targets (see later). Each intervention arm should contain at least 10 children in coma (Blantyre coma score ≤2).

**Group B: Severe acidosis**

Children will receive 40mls/kg of either Gelofusin or 4.5% human albumin solution. This will be given over the first hour following admission. However, the final volumes given will depend upon the attainment of pre-defined resuscitation targets (see later). Each intervention arm should contain at least 10 children in coma (Blantyre coma score ≤2).

The anticipated volumes are based upon the results of the previous pilot data^9^ and are identical to those given during the randomised trial^14^. However, in this study the final volume of fluids given to both study groups and for both intervention will ultimately depend upon the satisfactory attainment of resuscitation goals. The initial management and resuscitation targets are covered under the follow section.

# **Definition and treatment goals for children with hypovolaemic shock**

These have been adapted from the international consensus statement for paediatric shock^5^.

**Level one:** **Assessment for decreased perfusion and alteration of mental status (agitation, prostration or coma):** capillary refill > 2 seconds (cold shock) or flash capillary refill (warm shock), diminished (cold shock) or bounding (warm shock) peripheral pulses, mottled cool extremities (cold shock), or decreased urine output of 1 mL/kg/hr.

**Maintain airway, give oxygen and a**

**rapid infusion of 20mls/kg of colloid.**

Assessment at 30 minutes for level 1 goals of successful resuscitation

**Level 2: level one goals not achieved**

**Maintain airway and give oxygen.**

**Administer a further bolus of 20mls/kg of colloid**.

Assessment at 1 hour for level 1 goals of successful resuscitation.

(note: For children receiving > 20mls/kg of colloid we plan to insert a CVP line for the purposes of safety. The fixing of central line will not delay the administration of the second 20mls/kg of resuscitation, since this may be deleterious to the child.)

# **Level three: failure to achieve level one goals after 40mls/kg of colloid**

Assessment of CVP and perfusion pressure. For those with a CVP ≤8cm water or a perfusion pressure < threshold targets further bolus of colloid will be given ( in 10mls/kg aliquots) until CVP >8cm water or perfusion pressure threshold achieved and urine output > 1ml/kg/hr (level two goal).

**Level four: fluid refractory shock:** Failure to achieve level two goals after 80mls/kg, consider dopamine.

**Level one goals (all of the following)**

Heat rate within threshold range for age

Systolic blood pressure (BP) within threshold range for age

Capillary refill time <3secs

Oxygen saturations > 95% in room air

Table of the threshold heart rates, mean blood pressure and perfusion pressure (MAP-CVP) for age

| **Age (years)** | **Heart rate *(beats/min)** | **Systolic BP**  **(cm Hg)** | **Perfusion pressure (MAP-CVP)** |
| --- | --- | --- | --- |
| **Newborn** | 120–180 | 70 | 55 |
| **-1** | 120-180 | 75 | 60 |
| **-2** | 120-160 | 80 | 65 |
| **-7** | 100-140 | 85 | 65 |
| **-15** | 90-120 | 85 | 65 |

*should be only be assessed after the termination of a seizure

## Level two goals

CVP > 8 cm H20 or attainment of threshold perfusion pressure (see table)

plus urine output> 1mls/kg/hour.

**Randomisation procedure**

Randomisation will be conducted using a sealed card system. In the case of an emergency, where a child meets the clinical criteria for severe malaria but has evidence of profound shock (systolic blood pressure that is unrecordable or less < 80mm Hg) and volume resuscitation is required immediately, a children will be randomised before the receipt of the laboratory results and whilst consent is being obtained. The first 20mls/kg bolus will be given while the mother or guardian of the child is being consented and blood tests obtained. At this point if consent for the trial is refused or the malaria slide is negative for *P. falciparum* malaria parasites the child’s subsequent management will be as per local clinical protocol and by the attending clinicians’ judgement. The child will be dropped from the analysis of the study and the study number reallocated.

All children will be intensively monitored. The purpose of the intensive monitoring will be to establish the relative dose response and safety of each fluid regime required for correction of volume deficits and to monitor haemodynamic changes (in respiratory rate, oxygen saturation, heart rate, blood pressure and urine output) and clinical response induced by either colloid. To guide further management a central venous pressure line (CVP line) will be inserted into any child that develops refractory shock (failure to attain level one goals after one hour) or features suggestive of pulmonary oedema (development of bilateral fine, basal crepitations and falling oxygen saturations).

**Standard treatment of severe malaria**

All children with severe malaria are normally transferred to the high dependency unit. Standard management includes intravenous quinine infused in 5-10% dextrose, antibiotics (discontinued after negative blood cultures at 48 hours and normal lumbar puncture), antipyretics and anticonvulsants as per protocol. Hypoglycaemia is treated with a bolus of 2mls/kg of 25% dextrose. All children receive maintenance fluids (96mls/kg/day or 4mls/kg/hour of 5% dextrose/0.18% saline) but those with acidosis generally received intravenous volume expansion. Previous pilot studies have determined optimal volumes required. Children recruited into this study will receive only volume expansion by randomisation. At admission children will have blood taken for grouping and saved for cross-matching. Children with a haemoglobin < 5g/dl currently receive 20 mls/kg of whole blood.

# **d) Procedures**

Data collection and venous blood sampling will be in line with current, approved guidelines practiced on the KEMRI ward at Kilifi District Hospital. Details are listed below.

**i) Type of data to be collected and collection procedures to be followed.**

## Clinical procedures

The study will be conducted with continual monitoring of vital and neurological signs. Clinical assessment by members of the trial team will occur at 30 mins, 1 hour and followed by hourly (by the nursing personnel) until 8 hours post admission, and thereafter 4 hourly. One of the trial team will be attending to the child when they are receiving resuscitation fluids. Children will be managed according to the attainment of resuscitation goals described previously. The primary endpoints will be the correction of shock and volumes of colloid required to achieve this (ie: level one, level two etc).

**Potential complications**

The judicious use of intravenous fluids in children with a potential of raised intracranial pressure, cardiogenic shock or fluid overload is a concern. The study will be conducted with continual monitoring of vital and neurological signs. Further boluses of fluid will be withheld if vital and neurological signs indicate raised intracranial pressure (ICP) or heart failure. Raised ICP will be indicated by a rising blood pressure (>90% centile/age), together with a falling heart rate, the development of a sluggish reaction to or asymmetric pupillary reaction to light. Cardiac failure will be indicated by a worsening tachycardia and tachypnoea, de novo presence of fine crepitations in lung bases, deteriorating oxygen saturation and a sustained rise in CVP >15cm. These children will not receive any more fluid boluses and chest x-ray will be performed. Frusemide at 1mg/kg will be given to children in whom pulmonary oedema or right-sided heart failure is suspected or confirmed. In those with suspected raised ICP treatment with mannitol will be considered. The complications of a central venous pressure line include infection, blood loss and thrombosis. These will be minimised by using short CVP lines inserted into the femoral vein and advanced only 6-8 cm. They will remain in-situ for only up 48 hours then removed. The nurse in charge of the patient will only look after a maximum of two patients at one time.

**Study Monitoring**

A local data safety and monitoring board (DSMB) composed of clinical and scientifically qualified members will act as the main monitor for the trial, which will review all deaths life threatening complications (Dr Mike English, Dr Phillip Bejon and Bernhardt Ogutu). These monitors will assess each death and children developing neurological sequelae on a case by case basis. Otherwise we intend to provide the DSMB summary data after the first 40 children have been recruited.

#### Blood sampling and Laboratory procedures

Blood count, venous blood gases, blood sugar and chemistry (sodium, potassium and calcium) plasma creatinine, albumin, osmolality and red cell rheology will be measured at admission and 24hrs. Clotting and will be assessed at admission, 8 hours after colloid infusion and at 24 hours after admission (the details of the coagulation tests are covered in a separate proposal entitled ‘Investigation of coagulation activation in children with severe malaria’). Additional measurements of venous gas, plasma sodium, potassium, calcium, creatinine, albumin, red cell rheology and blood sugar will be taken at 8 hours to assess the response to volume resuscitation. Blood volumes taken for the purposes of monitoring are to be kept well within the maximum locally agreed volumes. In total 5mls of blood will be taken at admission (including all the standard blood tests) and an additional 2.5 mls the following 24 hours.

**9. DATA MANAGEMENT**

**(a) Data storage**

Original paper based data would be kept on file for 5 years from the completion of the proposed study. Patient data stored on computers is confidential and access restricted. Clinical and other data are backed up daily and copies of data files stored both on site and in the KEMRI/Wellcome Trust computer centre in Nairobi.

**b) Provisions for data verification and validation.**

Currently all data is directly entered in File-Maker Pro where most variables entered have dichotomous values only and the values for numerical fields are selected from a list. Initial and final diagnoses are also chosen from a list of common diagnoses. The programmes have been written to include logical and range checks and flags for the reduction of duplication errors. Appropriate information quality control procedures are already established. Children fulfilling the admission criteria for the study will be flagged by the computer to alert the admitting physician.

Descriptive statistics will be used to compare the clinical characteristics of the groups at baseline (admission), 30minutes, 1 hr, 4 hrs, 8 hrs, 12 hrs and at 24 hours, by intervention. The major outcomes of interest will be the resolution of clinical features of shock and major side effects or complications; development of abnormal clotting indices, pulmonary oedema and death (as dependant variables) between the different interventions (independent variables). The primary measure of this outcome will compare the proportions attaining level one goals for resuscitation in those receiving albumin and Gelofusin. Using ANOVA baseline (admission) haemodynamic parameters (heart rate, respiratory rate, oxygen saturation, mean blood pressure and coma score) will compared to those at 1 hr, 2 hours and 8 hours between the different intervention groups. For the purpose of analysis percentage mean change in the parameter will be used between the baseline and next time point.

Expected outcome: We have shown that albumin results in low mortality (< 5%), whilst saline was associated with an overall mortality ~20%, rising to 46% in those with coma. Historical control data from children with the same clinical criteria show mortality was between 30-50%, when volume expansion was not routine. If the dose response to Gelofusin is similar to albumin then assessment of the clinical safety of Gelofusin will take the above mortality data into consideration, as well as the evidence arising from the coagulation studies.

### 10. TIME FRAME

If ethical permission were granted it is proposed that the project begin in June 2004, and would continue for a maximum of 8 months. It is anticipated that a final report of these preliminary studies should be available by July 2005.

**11. ETHICAL CONSIDERATIONS.**

i) Do no harm. These children will be closely monitored and fluid boluses given cautiously. Further fluid boluses would be withheld from children showing signs of cardiogenic shock or fluid overload.

ii) Blood samples would be required from all study children. However, the volumes of blood required would be minimized wherever possible and be kept well within the maximum locally agreed volumes (10 mls per patient over the period of the study).

iii) Informed consent will be sought from all families involved in the proposed studies (see attached consent form).

iv) Information pertaining to children involved in the proposed studies would be kept on secure password protected databases. In addition, personal data would be indexed using unique identification numbers rather than personal names.

v) The well being of the sick child is paramount. Therefore, at any time the supervising physician can override the requirements of the study if there were a clinical deterioration.

**12. EXPECTED APPLICATION OF THE RESULTS**

## Benefits for the child

Continuous haemodynamic monitoring will enable us to conduct the study safely. The volumes of fluid given will be given cautiously and response monitored after each dose. Correction of hypovolaemia, has been shown to result in improved outcome. Studies of this nature are familiar to the personnel working on the high dependency unit at Kilifi; one to one nursing and a dedicated team of doctors will ensure that the child receives the best quality of clinical care. More detailed monitoring, for the potential complications, ensure that clinical treatment can be tailored to the hour-by-hour needs of the child.

## Benefits to the community

The results of this study will be the basis for the design of future trials of volume expansion. Demonstrating that a cheaper colloid is both safe and efficacious will allow more rational study design of future multicentre studies. If colloids were shown to reduce mortality, then future recommendations would be made to incorporate these cheaper solutions into standard management guidelines with for children with severe malaria.

# **13. ROLES OF THE INVESTIGATORS**

**Hannington Otieno, Sadik Mithwani, Richard Idro and Charles Newton** will be responsible for recruitment of the patients, monitoring and clinical care, which will be in the high dependency unit.

**Kathryn Maitland** is the Principal investigator who designed the study, will help with the conduct of the study and will perform the data analysis and interpretation.

14. **Budget**

Shillings Dollars

(a) Personnel, salaries & benefits disbursement. *no additional salaries required*

(b) Patient costs and/or supplies 143,750 2000

(c) Equipment *no additional equipment required*

(d) Travel and accommodation *none required*

(e) Transportation, vehicle repairs, insurance etc. *nil*

### (f) Operating expenses postage, printing etc 14,000 200

### (g) Animal Expenses *nil*

### (h) Consultancy Fees *nil*

### (i) Contigency funds (15% of a-e) 23,663 330

Total 181, 413 2530

This project is part of the KEMRI-Wellcome Trust collaborative programme which, is responsible for covering the clinical costs arising from this project.

###### 15. REFERENCES

1. Newton CR, Taylor TE, Whitten RO. Pathophysiology of fatal falciparum malaria in African children. *Am J Trop Med Hyg* 1998;**58**(5)**:**673-83.

2. Levin M, ed. Paediatric Emergencies. 2nd ed. London: Butterworths, 1987.

3. Severe falciparum malaria. World Health Organization, Communicable Diseases Cluster. *Trans R Soc Trop Med Hyg* 2000;**94 Suppl 1:**S1-90.

4. Herson VC, Todd JK. Prediction of morbidity in *Hemophilus influenzae* meningitis. *Pediatrics* 1977;**59**(1)**:**35-9.

5. Carcillo JA, Fields AI. Clinical practice parameters for hemodynamic support of pediatric and neonatal patients in septic shock. *Crit Care Med* 2002;**30**(6)**:**1365-78.

6. Carcillo JA, Davis AL, Zaritsky A. Role of early fluid resuscitation in pediatric septic shock. *Jama* 1991;**266**(9)**:**1242-5.

7. Dung NM, Day NP, Tam DT, et al. Fluid replacement in dengue shock syndrome: a randomized, double-blind comparison of four intravenous-fluid regimens. *Clin Infect Dis* 1999;**29**(4)**:**787-94.

8. Maitland K, Levin M, English M, et al. Severe P. falciparum malaria in Kenyan children: evidence for hypovolaemia. *Qjm* 2003;**96**(6)**:**427-34.

9. Maitland K, Pamba A, Newton CR, Levin M. Response to volume resuscitation in children with severe malaria. *Pediatr Crit Care Med* 2003;**4**(4)**:**426-31.

10. Emerson T. Unique features of albumin:A brief review. *Crit Care Med* 1989;**17**(7)**:**690-694.

11. Wilkes MM, Navickis RJ. Patient survival after human albumin administration. A meta-analysis of randomized, controlled trials. *Ann Intern Med* 2001;**135**(3)**:**149-64.

12. Vincent JL, Wilkes MM, Navickis RJ. Safety of human albumin--serious adverse events reported worldwide in 1998-2000. *Br J Anaesth* 2003;**91**(5)**:**625-30.

13. Human albumin administration in critically ill patients: systematic review of randomised controlled trials. Cochrane Injuries Group Albumin Reviewers. *Bmj* 1998;**317**(7153)**:**235-40.

14. Maitland K, Pamba A, English M, et al. Colloid infusion reduces the risk of death in children with severe malaria and acidosis: results of a randomised controlled trial of volume expansion with albumin or saline. *submitted* 2004.

15. Roberts J, Bratton S. Colloid Volume Expanders: Problems, pitfalls and possibilities. *Drugs* 1998;**55**(5)**:**621-630.

16. Nearman H, Herman M. Toxic effects of colloids in the intensive care unit. *Crit Care Clin* 1991;**7**(3)**:**713-723.

17. Salmon J, Mythen M. Pharmacology and physiology of the colloids. *Blood Rev* 1993;**7**(2)**:**114-120.

18. de Jonge E, Levi M. Effects of different plasma substitutes on blood coagulation:A comparative review. *Crit Care Med* 2001;**29**(6)**:**1261-1267.

19. Stockwell M, Stone N, Riley B. Colloid soultions in the critically ill: A randomized comparison of albumin and polygeline. *Anaesthesia* 1992;**47**(1)**:**3-6.

20. Clemens R, Pramoolsinsap C, Lorenz R, Pukrittayakamee S, Bock HL, White NJ. Activation of the coagulation cascade in severe falciparum malaria through the intrinsic pathway. *Br J Haematol* 1994;**87**(1)**:**100-5.

21. Hemmer CJ, Kern P, Holst FG, et al. Activation of the host response in human Plasmodium falciparum malaria: relation of parasitemia to tumor necrosis factor/cachectin, thrombin-antithrombin III, and protein C levels. *Am J Med* 1991;**91**(1)**:**37-44.

22. Management of the child with a serious infection or severe malnutrition. Geneva: World Health Organization, 2000.

23. Ladhani S, Lowe B, Cole AO, Kowuondo K, Newton CR. Changes in white blood cells and platelets in children with falciparum malaria: relationship to disease outcome. *Br J Haematol* 2002;**119**(3)**:**839-47.

24. Maitland K, Pamba A, English M, et al. Colloid infusion reduces the risk of death in children with severe malaria and acidosis: results of a Phase II randomised controlled trial of volume expansion with albumin or saline. *Lancet* 2004**:**submitted.

1. In a linked proposal : ‘Investigation of coagulation activation in children with severe malaria’ we will investigate the frequency of coagulation and endothelial activation in children with severe malaria. This will be conducted in concert with this study, and will enable us to describe changes in the coagulation activation consequent to volume expansion with albumin and Gelofusin. [↑](#footnote-ref-2)
