# Supplementary material for: Volume Expansion with Albumin Compared to Gelofusine in Children with Severe Malaria: Results of a Controlled Trial
Source: PLoS Clin Trials. 2006 Sep 15;1(5):e21. doi: 10.1371/journal.pctr.0010021 (PMC1569382; doi:10.1371/journal.pctr.0010021)
Supplement: Text S1 — (21 KB DOC) [file pctr.0010021.sd003.doc]

**Appendix**

## Design and findings of previous RCT

**Design**

This was an open randomised controlled trial, to compare the safety of resuscitation with albumin to saline in Kenyan children with severe malaria. All children received intravenous maintenance fluid (4% dextrose/0.18% saline) at a rate of 4mls/kg/hr and standard treatment. Patients were stratified into a moderately acidotic (MA) group (base deficit 8 to 15) and a severely acidotic (SA) group (base deficit >15). Based on the results of a pilot study in which we demonstrated that hypotension (systolic blood pressure < 80mmHg in children ≥ 1y and < 70mmHg in those <1 yr) complicated 39% of the severely acidotic group (base deficit > 15) at admission, both the trial committee, and external reviewers felt that withholding resuscitation fluids would be unacceptable in severely acidotic children. Thus, no control (maintenance only) arm was included in the SA group of the study; a control group was only in acceptable in the MA group. Children in the MA group were randomly assigned to one of three treatments: 4.5% albumin, 0.9% saline or control (maintenance only). Patients in the SA group were randomised to receive either 4.5% albumin or 0.9% saline. Saline and albumin boluses were given over the first hour following admission. Children with base deficits of 8 to 15 received 20 mls per kg, while those with base deficit >15 received up to 40 mls per kg. Rescue therapy (volume resuscitation with boluses of 20mls/kg of saline) was available for the control group in cases that developed decompensated shock (hypotension).

# Findings

Mortality was significantly more common in children assigned to saline 11/61 (18 %) than in children assigned to albumin 2/56 (3.6 %): relative risk 5.1, 95 % CI 1.2 to 22.8; P= 0.013. Most deaths occurred in children with severe acidosis: 8 of 25 (31 %) of those receiving saline as compared with 2 of 23 (9 %) in the albumin group (P = 0.06). Overall mortality in the moderate acidosis group was low, only 5/101 (5%) died (control 2/33 (6%); albumin 0/33 and saline 3/35 (9%)); however within hours of admission 5/33 (15%) of the control group required rescue therapy (volume resuscitation).

# Interpretation

This trial provided early evidence to suggest that albumin was superior resuscitation fluid than saline in children with severe malaria complicated by acidosis. The trial could not draw any firm conclusions about how either of these therapies compared to no bolus control, the standard of care across Africa, since the control group in this trial only included a lower risk group and power of the trial to demonstrate difference further constrained by the provision of rescue therapy in the control group. Owing to the high fatality in children with severe malaria complicated by acidosis the question of whether volume expansion is preferable to provision of maintenance fluids alone still needs to be addressed in definitive, adequately powered, studies before treatment recommendations can be made. The control group was therefore not included in the meta-analysis.
